# Site-Specific Responses to SERM Treatment in Postmenopausal Osteoporosis: No Clear Age Attenuation in a Real-World Study

**DOI:** 10.3390/medicina62071220

**Published:** 2026-06-23

**Authors:** Takashi Nagai, Eriko Hoshi, Koji Ishikawa, Koki Tsuchiya, Soji Tani, Yusuke Dodo, Keizo Sakamoto, Nobuyuki Kawate, Yoshifumi Kudo

**Affiliations:** 1 Department of Rehabilitation Medicine, School of Medicine, Showa Medical University, Shinagawa-ku, Tokyo 142-8666, Japan; 2 Department of Orthopedic Surgery, School of Medicine, Showa Medical University, Shinagawa-ku, Tokyo 142-8666, Japan; 3 Department of Rehabilitation, Yokohama Northern Hospital, Showa Medical University, Yokohama, Kanagawa 224-8503, Japan; 4 Department of Orthopaedic Surgery, Duke University, Durham, NC 27710, USA

**Keywords:** aging, bone mineral density, femoral neck, postmenopausal osteoporosis, selective estrogen receptor modulators

## Abstract

*Background*: Selective estrogen receptor modulators (SERMs) are widely used for postmenopausal osteoporosis, yet whether treatment response attenuates with aging in routine practice remains unclear. We examined age- and site-specific responses to SERM therapy. *Methods*: We retrospectively analyzed postmenopausal women with primary osteoporosis treated with a SERM for 1 year (2017–2021). Participants were stratified by age (50–64, 65–74, and ≥75 years). We evaluated changes in bone mineral density (BMD) at the lumbar spine (L2–4) and femoral neck and changes in urinary NTX and serum BAP. Multivariable linear regression modeled BMD change ratios (1-year/baseline) adjusting for baseline site-specific BMD, estimated glomerular filtration rate (eGFR), and active vitamin D co-therapy (none, alfacalcidol, or eldecalcitol). The primary endpoint was the 1-year change in lumbar spine BMD; secondary endpoints included femoral neck BMD and bone turnover markers. *Results*: Lumbar spine BMD increased significantly across all age groups, whereas femoral neck BMD increased significantly only in women aged 50–64 years. However, BMD change ratios did not differ among age groups at either site. In adjusted models, age was not independently associated with BMD change at the lumbar spine or femoral neck. Lower baseline BMD predicted larger relative gains at both sites, and eldecalcitol co-therapy was independently associated with femoral neck BMD response. *Conclusions*: In real-world practice, BMD changes observed during SERM treatment were site-specific rather than clearly age-dependent. Lumbar spine BMD improved across age groups, whereas femoral neck changes were smaller and less consistent.

## 1. Introduction

The global prevalence of osteoporosis continues to increase, and osteoporosis-related healthcare costs are projected to reach USD 25.3 billion annually in the United States by 2025 [1,2]. Approximately one in two adult women and one in five men will experience at least one fragility fracture during their lifetime [3], underscoring the growing importance of effective prevention and treatment strategies for osteoporosis [4].

Currently available pharmacological treatments for osteoporosis include parathyroid hormone (PTH) analogs [5], anti-sclerostin antibodies [6], anti-receptor activator of nuclear factor-kappa B ligand (RANKL) antibodies [7], and bisphosphonates [8]. Selective estrogen receptor modulators (SERMs) represent another class of anti-osteoporotic agents that exert tissue-selective estrogen receptor agonist or antagonist effects [9].

Estrogen plays a crucial role in maintaining bone mass and preventing osteoporosis [10] and has historically been used as part of hormone replacement therapy in postmenopausal women. However, systemic estrogen therapy is associated with non-skeletal adverse effects, including increased risks of breast cancer and endometriosis [11]. SERMs were developed to minimize these risks by selectively targeting estrogen receptors in bone while limiting stimulation of the uterus and breast tissue [12]. Raloxifene, a benzothiophene derivative without a steroidal backbone [13], and bazedoxifene, an indole-based compound [14], are representative SERMs currently used in clinical practice. Raloxifene has been shown to inhibit bone resorption and increase bone mass without stimulating the endometrium [10].

Despite their demonstrated skeletal effects, SERMs are not recommended for the treatment of osteoporosis in the American College of Physicians (ACP) guidelines [15]. This recommendation is based on evidence from randomized controlled trials indicating higher rates of treatment discontinuation due to adverse events compared with placebo, as well as observational data showing an increased risk of venous thromboembolism [16]. Nevertheless, SERMs continue to be prescribed in clinical practice, particularly for selected postmenopausal women in whom other anti-osteoporotic agents are contraindicated or poorly tolerated, highlighting the need to clarify their real-world BMD responses across different age groups. In contrast, clinical guidelines in the United Kingdom recommend raloxifene as a treatment option [4], and raloxifene and bazedoxifene are listed as treatment options in U.S. and Japanese clinical guidelines [17,18]. Previous studies have demonstrated that SERM therapy increases bone mineral density (BMD) and suppresses bone turnover markers in postmenopausal women [19], and a multivariate subanalysis of the Multiple Outcomes of Raloxifene Evaluation (MORE) study reported that the skeletal effects of raloxifene were independent of age [20].

Estrogen receptor expression is influenced by circulating estrogen levels [21]. Estrogen levels begin to decline after approximately 40 years of age, leading to reduced estrogen receptor expression in osteocytes [22,23] and bone tissue [22]. Given that SERMs exert their pharmacological effects through estrogen receptor modulation [10], it is biologically plausible that their skeletal efficacy may be attenuated in older individuals. In addition, the probability of major osteoporotic fractures estimated using the Fracture Risk Assessment Tool (FRAX) increases with age [24], and the incidence of secondary fractures following vertebral fractures is also higher in older populations [25]. However, age-related differences in the clinical efficacy of SERMs in real-world settings remain insufficiently characterized.

Therefore, this study aimed to investigate the effects of SERM therapy on BMD and bone turnover markers in postmenopausal women with primary osteoporosis stratified by age, with a particular focus on potential age- and site-specific differences in treatment response.

## 2. Materials and Methods

### 2.1. Subjects

Female patients diagnosed with postmenopausal primary osteoporosis who were treated with selective estrogen receptor modulators (SERMs) at our outpatient clinic for at least 1 year between 2017 and 2021 were included in this retrospective study.

Regarding pharmacological treatment, both raloxifene and bazedoxifene were prescribed during the study period (2017–2021). Raloxifene tended to be used more frequently in the earlier years, whereas bazedoxifene gradually became the predominant agent in later years; however, the two agents overlapped throughout the study period.

Primary postmenopausal osteoporosis was diagnosed according to established diagnostic criteria for primary osteoporosis, based on bone mineral density and/or the presence of fragility fractures [18]. In brief, patients were considered to have osteoporosis when the BMD T-score at the lumbar spine or femoral neck was ≤−2.5, or when a fragility fracture was present in association with low bone mass. Patients with suspected secondary osteoporosis or other metabolic bone diseases were not included in the target population.

### 2.2. Exclusion Criteria

Patients younger than 50 years of age and those who did not undergo bone mineral density (BMD) assessment during the study period were excluded. Patients who declined consent for the use of their clinical data for research purposes were also excluded.

### 2.3. Patient Classification by Age

Participants were stratified into three age groups according to chronological age at baseline: 50–64 years, 65–74 years, and 75 years and over.

### 2.4. BMD Analysis

Bone mineral density (BMD) was assessed at baseline and after 1 year of SERM therapy. BMD measurements were obtained at the lumbar spine (anteroposterior L2–4) and femoral neck using dual-energy X-ray absorptiometry (DXA) (Discovery DXA System; Hologic, Inc., Marlborough, MA, USA).

### 2.5. Measurement of Bone Turnover Markers

Bone turnover markers were assessed using urinary type I collagen cross-linked N-telopeptide (NTX) and serum bone-specific alkaline phosphatase (BAP). Urinary NTX was measured using samples obtained from the second voided urine in the early morning. Patients were provided with urine collection containers in advance and were instructed to bring the second morning urine sample on the day of assessment. Serum BAP was measured from blood samples collected at any time of day.

Urinary NTX levels were determined using the Vitros NTX assay for type I collagen cross-linked N-telopeptide (Ortho Clinical Diagnostics, Tokyo, Japan).

### 2.6. Fractures

Pre-existing fractures, including vertebral and proximal femoral fractures, were assessed at baseline before initiation of SERM therapy. Incident fractures involving any thoracic vertebra, lumbar vertebra, or the femoral neck were evaluated during the first year after SERM administration.

### 2.7. Concomitant Therapy and Lifestyle Intervention

All patients were prescribed a SERM, with or without concomitant active vitamin D_3_. Patients with reduced renal function were prescribed alfacalcidol, whereas those with preserved renal function were prescribed eldecalcitol. Patients who declined active vitamin D_3_ therapy because they were already taking vitamin D supplements or regularly consuming vitamin D-rich foods received SERM monotherapy.

In addition to pharmacological treatment, all patients were instructed to perform dynamic flamingo exercises (open-eyed single-leg standing) three times daily for 60 s per session [26]. To ensure adequate endogenous vitamin D production, patients were also advised to obtain at least 10 min of sun exposure per day during summer and 30 min per day during winter [27].

### 2.8. Reporting Guidelines

This study was conducted and reported in accordance with the Strengthening the Reporting of Observational Studies in Epidemiology (STROBE) statement [28].

### 2.9. Statistical Analysis

Statistical analyses were performed using Stat Flex version 7.0.10 (2020) unless otherwise specified. Linear mixed-effects models were fitted in R version 4.5.2 (R Foundation for Statistical Computing, Vienna, Austria) using the lme4 and lmerTest packages. All tests were two-sided, and *p* < 0.05 was considered statistically significant.

The primary endpoint was the 1-year change in lumbar spine BMD. Secondary endpoints included the 1-year change in femoral neck BMD and changes in bone turnover markers (urinary NTX and serum BAP). Both absolute changes (1-year minus baseline) and relative changes (1-year/baseline) were evaluated.

Baseline characteristics were compared among age groups using one-way ANOVA or the Kruskal–Wallis test, as appropriate. Categorical variables were compared using the chi-square test.

For longitudinal BMD outcomes, we used a factorial repeated-measures framework implemented as a linear mixed-effects model with a participant-level random intercept. Fixed effects included time (baseline vs. 1 year), skeletal site (lumbar spine vs. femoral neck), age group, and their interaction terms. The time × site × age group interaction tested whether age group modified site-specific BMD changes over 1 year. The model was additionally adjusted for baseline site-specific BMD, estimated glomerular filtration rate (eGFR), serum albumin, and skeletal muscle index.

Within-group pre–post comparisons were presented descriptively using the paired *t*-test or Wilcoxon signed-rank test. Bone turnover markers were analyzed descriptively using the Wilcoxon signed-rank test due to non-normal distributions. Group comparisons of relative changes among age groups were performed using Dunn’s test with Bonferroni correction. Absolute BMD changes are provided as supportive analyses (Table S2).

In multivariable linear regression analyses of BMD change ratios, active vitamin D co-therapy was treated as a three-level categorical variable (monotherapy, alfacalcidol, eldecalcitol) using two dummy variables with monotherapy as the reference. Sensitivity analyses additionally included SERM type (bazedoxifene vs. raloxifene).

Analyses were performed using complete-case data with no imputation; 20 participants were excluded from multivariable analyses due to missing eGFR values. Sample size estimation was conducted using G*Power (version 3.1) assuming an effect size of 0.5, α = 0.05, and power = 0.95, requiring at least 47 participants per age group.

### 2.10. Ethics

This study was conducted in accordance with the Declaration of Helsinki and the ethical guidelines for medical research involving human subjects. The study protocol was reviewed and approved by the Ethics Committee on Research Involving Human Subjects at the School of Medicine, Showa University (approval no. 2024-219-A).

Given the retrospective nature of the study, the requirement for informed consent was waived by the ethics committee.

## 3. Results

### 3.1. Patient Background

**Figure 1:**
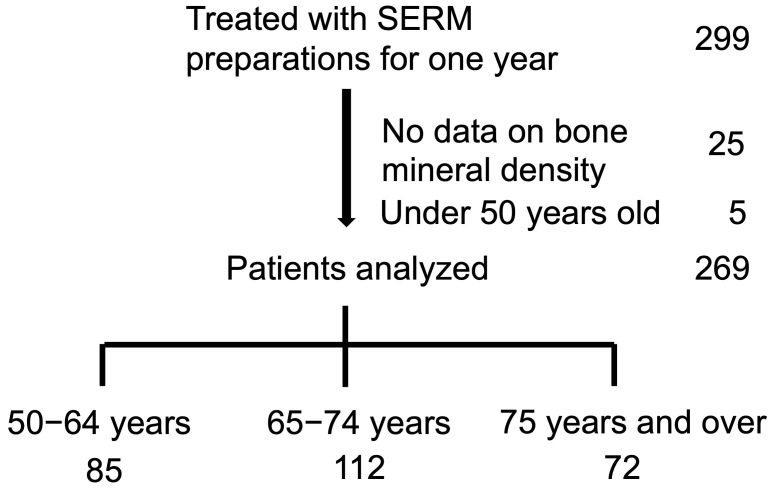
Overview of study protocol.

A total of 299 patients were initially enrolled. Among these, 25 patients with missing data and five patients younger than 50 years were excluded, leaving 269 patients for the final analysis. The mean age of the included patients was 68.8 ± 8.5 years. Of these, 85 patients were aged 50–64 years, 112 were aged 65–74 years, and 72 were aged 75 years and over (Figure 1). Prior osteoporosis treatments were categorized as no medication, active vitamin D_3_, bisphosphonates, denosumab, parathyroid hormone (PTH), or romosozumab. The distribution of prior medications and detailed baseline characteristics of the study population are summarized in Table 1.

Of the 299 patients prescribed SERM therapy, 269 were included in the final analysis after excluding five patients younger than 50 years and 25 patients with missing bone mineral density data. For multivariable regression analyses, 249 participants were included because eGFR was missing in 20 of the 269 eligible patients.

### 3.2. Existing Fractures

Existing fractures were observed in 40% of the patients included in the study. The prevalence of existing fractures increased with age, affecting 25% of patients aged 50–64 years, 44% of those aged 65–74 years, and 51% of those aged 75 years and over. Existing proximal femoral fractures were identified in four patients, accounting for 1% of the overall study population (Table 1).

### 3.3. New Fractures

Overall, 13 incident fractures were observed during the 1-year follow-up period. These included one fracture in the 50–64-year group (one vertebral fracture), six fractures in the 65–74-year group (all vertebral fractures), and six fractures in the 75 years and over group (five vertebral fractures and one proximal femoral fracture) (Table 1).

The overall incidence of new vertebral fractures was 4.5%. When stratified by age, new vertebral fractures occurred in 1.2% of patients aged 50–64 years, 5.4% of those aged 65–74 years, and 8.3% of those aged 75 years and over, demonstrating an age-related increase in the incidence of new fractures.

### 3.4. SERMs and Concomitant Active Vitamin D_3_

Among the two SERMs used, raloxifene was administered to 71 patients (26%), whereas bazedoxifene was administered to 198 patients (74%) (Table 2). Overall, 202 patients (75%) received concomitant eldecalcitol, 41 patients (15%) received alfacalcidol, and 26 patients (10%) received SERM monotherapy (Table 2).

When stratified by age group, there were no significant differences in the distribution of patients receiving eldecalcitol, alfacalcidol, or SERM monotherapy, nor in the proportions of patients treated with raloxifene or bazedoxifene (Table 2).

### 3.5. BMD

**Figure 2:**
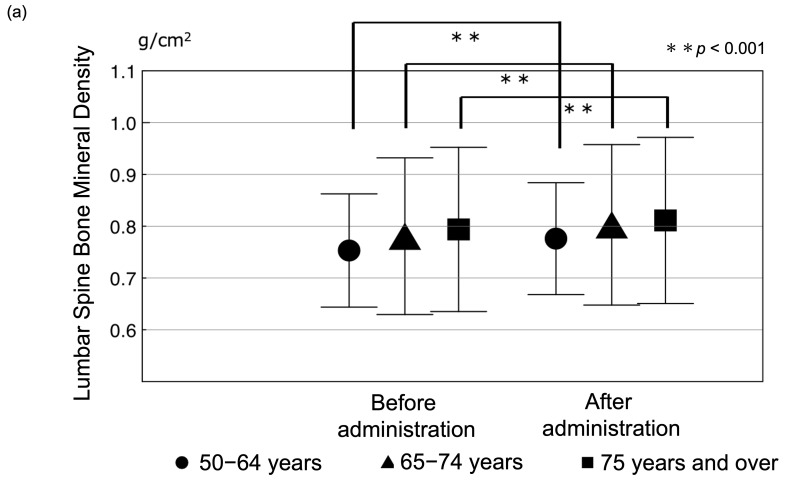

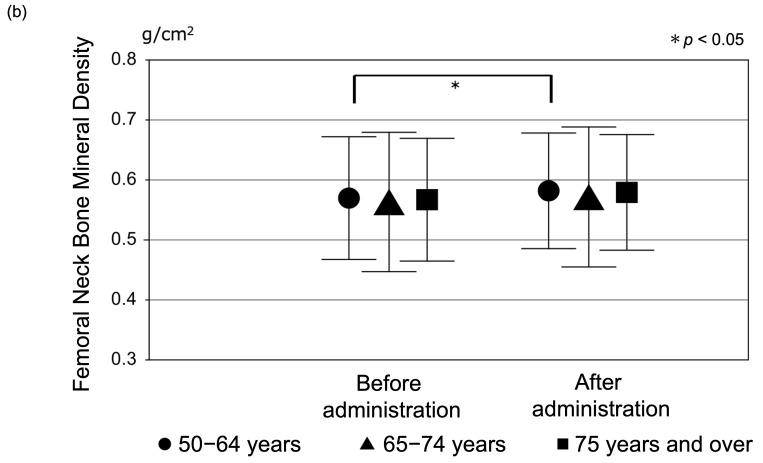
Changes in bone mineral density (BMD) following SERM treatment. (**a**) Lumbar spine BMD increased significantly at 1 year after SERM administration in the 50–64, in the 65–74 and 75 years and over groups (*p* < 0.001 for all groups). (**b**) Femoral neck BMD increased significantly only in the 50–64 years group (*p* < 0.05), whereas no significant changes were observed in the 65–74 or 75 years and over groups.

Lumbar spine BMD increased significantly at 1 year compared with baseline in all age groups (all *p* < 0.001; Figure 2a; Table 3). Femoral neck BMD showed a modest change; within-group analyses indicated a statistically significant increase only in the 50–64 years group, whereas changes were not statistically significant in the 65–74 and 75 years and over groups (Figure 2b; Table 3). Although the total cohort showed a statistically significant increase in femoral neck BMD, this overall change was mainly driven by the 50–64-year group; changes in the 65–74-year and ≥75-year groups did not reach statistical significance in within-group analyses.

However, the relative changes (ratios) in BMD over 1 year did not differ among age groups at either skeletal site (Table 4). Moreover, in a linear mixed-effects model adjusting for baseline BMD, eGFR, albumin, and SMI, the three-way interaction (time × site × age group) was not significant (*p* = 0.887), providing no evidence that age group modified site-specific BMD changes over 1 year.

Absolute changes in BMD (1-year minus baseline) are provided in Supplementary Table S2.

### 3.6. Bone Metabolism Markers

Levels of the bone turnover markers urinary NTX and serum BAP decreased significantly in all age groups at 1 year after SERM administration compared with baseline (Table 3). There were no significant differences in the relative changes (ratios) in either marker among age groups (Table 4).

### 3.7. Multivariable Analysis

In multivariable linear regression analyses adjusting for baseline age, baseline eGFR, baseline site-specific BMD, and concomitant active vitamin D co-therapy modeled as a three-group categorical variable with SERM monotherapy (no active vitamin D) as the reference (Table 5), baseline age was not independently associated with the 1-year BMD change ratio at either the lumbar spine or femoral neck.

For the lumbar spine, baseline lumbar spine BMD was significantly and inversely associated with the lumbar spine BMD change ratio (*p* = 0.00050), whereas neither eldecalcitol nor alfacalcidol co-therapy (each vs. SERM monotherapy) showed significant associations. The model explained 5.9% of the variance in the lumbar spine BMD change ratio (R^2^ = 0.059; adjusted R^2^ = 0.040).

For the femoral neck, baseline femoral neck BMD was significantly and inversely associated with the femoral neck BMD change ratio (*p* < 0.001). In addition, eldecalcitol co-therapy (vs. SERM monotherapy) was independently associated with the femoral neck BMD change ratio (*p* = 0.04254), whereas alfacalcidol co-therapy was not. The model explained 16.2% of the variance in the femoral neck BMD change ratio (R^2^ = 0.162; adjusted R^2^ = 0.145).

#### Sensitivity Analyses

After additionally adjusting for SERM type (bazedoxifene vs. raloxifene), the main findings were unchanged (Supplementary Table S1).

### 3.8. Complications

No cases of venous thromboembolism (VTE), cerebral infarction, osteonecrosis of the jaw, or atypical femoral fractures were observed during the 1-year observation period. However, the study was not designed or powered to evaluate rare adverse events.

## 4. Discussion

Principal findings. In this retrospective real-world cohort of postmenopausal women with osteoporosis treated with SERMs, we evaluated whether the 1-year treatment response differed by age and skeletal site. Three key findings emerged. First, the relative 1-year change in BMD did not differ among age groups at either the lumbar spine or femoral neck. Second, lumbar spine BMD increased significantly in all age groups, whereas femoral neck BMD increased significantly only in women aged 50–64 years; nevertheless, femoral neck BMD was broadly maintained in the older groups. Third, after adjustment for baseline BMD, renal function, and active vitamin D preparation, chronological age was not an independent predictor of BMD change at either site. Collectively, these results indicate that SERM response is primarily site-specific rather than age-dependent, supporting the use of SERMs in older patients when vertebral-oriented BMD maintenance is a key objective.

Biological plausibility of site-specific response. The observed site-specific pattern is biologically plausible. The lumbar spine is trabecular-rich and metabolically active, whereas the femoral neck has a higher cortical component and may show smaller short-term changes with antiresorptive therapy, particularly in advanced age. Beyond the trabecular–cortical composition, site-specific turnover and microenvironmental differences may contribute. The lumbar spine generally exhibits higher remodeling activity and may therefore show more detectable short-term changes with antiresorptive therapy, whereas the femoral neck has a larger cortical component with slower turnover. In addition, age-related changes such as osteocyte senescence and increased marrow adiposity may attenuate responsiveness at cortical-rich sites, potentially contributing to the smaller and less consistent femoral neck response in older patients [29,30,31].

Determinants of response beyond age. Beyond age, baseline BMD was the strongest determinant of relative response at both sites, with lower baseline BMD associated with larger proportional gains. Additionally, eldecalcitol co-therapy (vs. no active vitamin D) was independently associated with femoral neck BMD response, whereas alfacalcidol was not. This may reflect differences in pharmacological potency and fracture prevention evidence between active vitamin D analogs, as eldecalcitol has demonstrated stronger anti-fracture efficacy than alfacalcidol in prior studies [32], and has been reported to have more favorable effects on bone microstructure [33].

Alternatively, this finding may partially reflect confounding by indication: in our clinic, alfacalcidol was preferentially prescribed to patients with reduced renal function, and lower renal function may be associated with poorer skeletal response regardless of the anti-osteoporotic regimen. In the multivariable models, eGFR showed a borderline association with femoral neck BMD response (*p* ≈ 0.06), suggesting a potential renal function-related influence. Because this study was not designed to isolate the causal effect of vitamin D analog selection, these results should be interpreted cautiously as associations in routine practice.

Comparison with prior evidence and guideline context. Our findings that chronological age was not independently associated with the 1-year BMD response are consistent with prior evidence suggesting that the skeletal effects of raloxifene are largely independent of age [20]. In clinical practice, SERMs remain relevant because treatment selection is often constrained by comorbidity, tolerability, and patient preference. While the ACP guideline does not recommend SERMs for routine osteoporosis treatment, largely due to discontinuation and safety considerations, particularly venous thromboembolism risk [15,16], other guidelines (e.g., the UK guideline) include raloxifene as a treatment option in selected patients [4]. In this context, our real-world data support SERMs as a practical option when vertebral-oriented BMD maintenance is prioritized.

Interpretation in the setting of age-related endocrine changes. Although estrogen receptor expression may decline with age and lower circulating estrogen could theoretically attenuate SERM efficacy [21,22,23], the preserved lumbar spine response observed across age groups suggests that antiresorptive effects at trabecular-rich sites may remain clinically meaningful even in advanced age.

Clinical implications. Clinically, these findings reinforce that treatment selection should be individualized by skeletal site and patient profile. SERMs are not “high-potency” BMD-raising agents compared with anabolic therapies or denosumab, but they can contribute to vertebral-oriented BMD improvement/maintenance with convenient oral administration, which is often valued in older outpatients. From a clinical perspective, the magnitude of the observed lumbar spine BMD increase over 1 year was modest (approximately 2–3% on average) but may still be meaningful for vertebral-oriented management in routine practice. While BMD change is a surrogate outcome and does not directly translate into fracture risk reduction in this uncontrolled study, these changes are broadly in the range reported for antiresorptive therapies. Therefore, SERMs may remain a pragmatic option when treatment selection is constrained by comorbidities, tolerability, or patient preference, particularly when the primary goal is maintenance or modest improvement of vertebral BMD.

Strengths and limitations. The strengths of this study include the size of the cohort, routine-practice setting, and the combined use of site-specific regression and longitudinal mixed-effects modeling. Treatment selection bias is an inherent limitation of this retrospective, uncontrolled study. In routine practice, physicians may preferentially prescribe SERMs to patients perceived as less suitable for other anti-osteoporotic agents (e.g., gastrointestinal intolerance or adherence concerns with oral bisphosphonates, concerns regarding hypocalcemia risk or injection preference for denosumab, or broader considerations of comorbidity and frailty). Therefore, the enrolled older patients may have differed in baseline health status and fracture risk compared with patients treated with other agents, which may limit generalizability. Accordingly, our findings should be interpreted as BMD changes observed during SERM treatment rather than definitive causal treatment effects. Several limitations warrant consideration. The retrospective design limits causal inference and treatment selection was not randomized. Although both raloxifene and bazedoxifene overlapped throughout the study period, prescribing patterns shifted over calendar time; therefore, residual confounding by indication and/or time trends cannot be fully excluded. The number of incident fractures was small, and the study was not powered to evaluate fracture outcomes. Urinary NTX was obtained using the second morning void, whereas serum BAP was measured from samples collected at varying times of day; this may introduce random measurement variability, likely biasing observed changes toward the null. Because baseline BMD was inversely associated with relative BMD change, regression to the mean may have contributed to larger apparent proportional gains among participants with lower baseline values; therefore, observed changes should be interpreted cautiously in this uncontrolled observational setting.

Safety remains important when prescribing SERMs. Although no VTE events were observed during the 1-year follow-up in this cohort, the absence of events in a single-center observational study with limited follow-up does not negate the established VTE risk associated with SERMs. Therefore, individualized risk assessment and clinical vigilance remain essential, particularly in older patients [34].

## 5. Conclusions

In this real-world cohort, lumbar spine BMD increased across all age groups, whereas femoral neck BMD showed a significant within-group increase only in women aged 50–64 years but was broadly maintained in older groups. Chronological age was not independently associated with BMD change after adjustment, and no clear age-related attenuation of site-specific BMD changes was observed. These findings support SERMs as a pragmatic option in selected older patients when treatment goals focus on vertebral-oriented BMD maintenance, while acknowledging the limitations of an uncontrolled retrospective design.

## Figures and Tables

**Table 1:** Patient parameters as per the three age groups evaluated in this study.

	Total	50–64 Years	65–74 Years	75 Years and over	*p * Value
Number of patients	269	85	112	72	
Age (years)—Mean (SD)	68.8(8.5)	58.9 (3.5)	69.7 (2.9)	79.2 (3.9)	*p* < 0.001
Height (cm)—Median [IQR]	153.3 [149.5–156.6]	155.6 [153.1–158.2]	153.2 [149.6–156.4]	149.8 [144.0–153.2]	*p* < 0.001
Weight (kg)—Mean (SD)	51.0 (8.2)	50.6 (7.5)	51.6 (8.6)	50.6 (8.3)	0.55
BMI (kg/m^2^)—Mean (SD)	21.9 (3.3)	20.8 (2.7)	22.2 (3.4)	22.8 (3.5)	*p* < 0.001
Albumin (g/dL)—Mean (SD)	4.3 (0.3)	4.4 (0.3)	4.2 (0.3)	4.2 (0.3)	*p* < 0.001
Serum calcium level (mg/dL)—Median [IQR]	9.4 [9.1–9.6]	9.4 [9.2–9.7]	9.4 [9.1–9.6]	9.3 [8.5–9.1]	0.24
eGFR—Mean (SD)	71.8 (20.0)	79.2 (17.3)	70.9 (22.2)	64.1 (15.9)	*p* < 0.001
PTH-intact (pg/mL)—Mean (SD)	41.2 (23.9)	39.4 (18.9)	44.7 (29.9)	40.6 (18.9)	0.69
L-Spine BMD (g/cm^2^)—Mean (SD)	0.776 (0.142)	0.753 (0.109)	0.784 (0.151)	0.791 (0.159)	0.10
Femoral neck BMD (g/cm^2^)—Mean (SD)	0.561 (0.112)	0.570 (0.102)	0.562 (0.116)	0.550 (0.116)	0.56
NTX (nMBCE/mMCr)—Median [IQR]	48.8 [32.4–71.6]	56.0 [40.6–76.9]	47.7 [32.6–72.1]	42.6 [5.5–66.0]	*p* < 0.05
BAP (U/L)—Median [IQR]	14.1 [10.9–18.2]	14.9 [11.5–18.4]	14.1 [11.5–18.2]	13.1 [10.0–17.9]	0.23
Existing fractures					
Any (%)	107 (40.0)	21 (24.7)	49 (43.8)	37 (51.4)	
Thoracic and lumbar vertebral fracture (%)	97 (36.1)	14 (16.5)	46 (41.1)	37 (51.4)	*p* < 0.01
Femoral neck fracture (%)	4 (1.5)	3 (3.5)	1 (1.0)	0 (0)	
Fractures occurring during the year					
Any (%)	13 (4.8)	1 (1.2)	6 (5.4)	6 (8.3)	
Thoracic vertebral fracture (%)	7 (2.6)	1 (1.2)	4 (3.6)	2 (2.8)	0.58
Lumbar vertebral fracture (%)	5 (1.9)	0 (0)	2 (1.8)	3 (4.2)	
Femoral neck fracture (%)	1 (0.4)	0 (0)	0 (0)	1 (1.4)	
Prior treatment					
Treatment Naive	184	64	79	41	*p* < 0.05
Active vitamin D	52	16	23	13	
Bisphosphonate	22	4	7	11	
PTH/denosumab/ romosozumab	11	1/0/0	0/2/1	4/3/0	

SD, standard deviation; IQR, interquartile range; eGFR, estimated glomerular filtration rate; PTH, intact parathyroid hormone; NTX, cross-linked N-telopeptide of type 1 collagen; nMBCE/mMCr, nmol bone collagen equivalents/mmol creatinine; BAP, bone alkaline phosphatase.

**Table 2:** Number of patients who received raloxifene and bazedoxifene.

	Total	50–64 Years	65–74 Years	75 Years and over	*p* Value
Raloxifene	71	24	28	19	
+Eldecalcitol	45	18	18	9	0.19
+Alfacalcidol	15	4	7	4	
Single-drug administration	11	2	3	6	
Bazedoxifene	198	61	84	53	
+Eldecalcitol	157	52	66	39	0.15
+Alfacalcidol	26	3	12	11	
Single-drug administration	15	6	6	3	
Raloxifene or bazedoxifene (%)	269 (100)	85 (31.6)	112 (41.6)	72 (26.8)	
+Eldecalcitol (%)	202 (75)	70	84	48	0.16
+Alfacalcidol (%)	41 (15)	7	19	15	
Single-drug administration (%)	26 (10)	8	9	9	

For evaluating the effect of single-drug administration and SERM plus alfacalcidol or eldecalcitol by age group, χ-square test was used.

**Table 3:** Lumbar spine and femoral neck BMD and bone turnover markers at baseline and 1 year after SERM therapy, stratified by age group.

	**Lumbar Spine BMD (g/cm^2^)**			**Femoral Neck BMD (g/cm^2^)**		
	**Before Administration**	**One Year After Administration**	***p* Value**	**Before Administration**	**One Year After Administration**	***p* Value**
Total Mean (SD)	0.776 (0.142)	0.797 (0.144)	*p* < 0.001	0.561 (0.112)	0.571 (0.110)	*p* < 0.01
50–64 years Mean (SD)	0.753 (0.109)	0.776 (0.108)	*p* < 0.001	0.570 (0.102)	0.582 (0.096)	*p* < 0.05
65–74 years Mean (SD)	0.784 (0.151)	0.805 (0.155)	*p* < 0.001	0.562 (0.116)	0.570 (0.117)	0.09
75 years and over Mean (SD)	0.791 (0.159)	0.809 (0.160)	*p* < 0.001	0.550 (0.116)	0.560 (0.116)	0.18
	**Urine NTX (nMBCE/mMCr)**			**Serum BAP (U/L)**		
	**Before Administration**	**One Year After Administration**	***p*** ** Value**	**Before** **Administration **	**One Year After Administration**	***p** * **Value**
Total Median [IQR]	48.8 [32.4–71.6]	28.6 [21.1–39.7]	*p* < 0.001	14.1 [10.9–18.2]	10.3 [8.4–12.8]	*p* < 0.001
50–64 years Median [IQR]	56.0 [40.6–76.9]	28.0 [18.9–39.9]	*p* < 0.001	14.9 [11.5–18.4]	10.2 [8.2–12.5]	*p* < 0.001
65–74 years Median [IQR]	48.3 [33.2–72.4]	29.0 [21.9–39.5]	*p* < 0.001	14.1 [11.5–18.2]	10.6 [8.5–13.1]	*p* < 0.001
75 years and over Median [IQR]	42.6 [25.5–66.0]	30.4 [21.7–41.3]	*p* < 0.001	13.1 [10.0–17.9]	9.9 [8.4–12.6]	*p* < 0.001

Bone density of lumbar spine and femoral neck were analyzed using the paired Student’s *t*-test, whereas urine NTX and serum BAP were analyzed using Wilcoxon signed-rank test. SD: standard deviation; IQR: interquartile range; BMD: bone mineral density; NTX: cross-linked N-telopeptide of type 1 collagen; nMBCE/mMCr: nmol bone collagen equivalents/mmol creatinine; BAP: bone alkaline phosphatase.

**Table 4:** Relative changes (ratios) in lumbar spine and femoral neck BMD and bone turnover markers over 1 year, and comparisons among age groups.

	(a) 50–64 Years	(b) 65–74 Years	(c) 75 Years and over	*p* Value
Lumbar Spine BMD (ratio) Median [IQR]	1.03 [1.01–1.06]	1.03 [1.00–1.05]	1.02 [1.00–1.06]	a–b 1.00; b–c 1.00; a–c 0.39
Femoral Neck BMD (ratio) Median [IQR]	1.03 [0.98–1.07]	1.01 [0.97–1.05]	1.02 [0.97–1.04]	a–b 0.64; b–c 1.00; a–c 0.73
NTX (ratio) Median [IQR]	0.57 [0.39–0.75]	0.56 [0.45–0.87]	0.62 [0.46–0.95]	a–b 0.92; b–c 0.49; a–c 0.08
BAP (ratio) Median [IQR]	0.72 [0.54–0.87]	0.72 [0.59–0.89]	0.71 [0.59–1.01]	a–b 1.00; b–c 1.00; a–c 0.83

Data are presented as the median [IQR]. Relative changes are expressed as the ratio of the 1-year value to the baseline value (1-year/baseline). Pairwise comparisons were performed using Dunn’s test with Bonferroni correction. IQR, interquartile range; BMD, bone mineral density; NTX, cross-linked N-telopeptide of type I collagen; BAP, bone alkaline phosphatase.

**Table 5:** Multivariable linear regression analyses of factors associated with 1-year BMD change ratios (1-year/baseline) at the lumbar spine and femoral neck after SERM therapy.

**A-1. Lumbar Spine BMD Change Ratio**				
**Variable**	**β**	**SE(β)**	**stdβ**	***p* Value**
Baseline Age (years)	−2.666 × 10^−5^	4.327 × 10^−4^	−0.0041	0.95092
Baseline eGFR	−8.732 × 10^−6^	1.824 × 10^−4^	−0.0032	0.96186
Baseline Lumbar Spine BMD	−0.08820	0.02499	−0.2266	*p* < 0.001 **
Alfacalcidol (vs. monotherapy)	−4.951 × 10^−4^	0.01631	−0.0030	0.97582
Eldecalcitol (vs. monotherapy)	0.01569	0.01349	0.1139	0.24590
Model fit: R^2^ = 0.059, Adjusted R^2^ = 0.040, *n* = 249				
**A-2. Femoral Neck BMD Change Ratio**				
**Variable**	**β**	**SE(β)**	**stdβ**	***p** * **Value**
Baseline Age (years)	2.118 × 10^−4^	8.713 × 10^−4^	0.0152	0.80817
Baseline eGFR	−7.010 × 10^−4^	3.681 × 10^−4^	−0.1189	0.05801
Baseline Femoral Neck BMD	−0.4034	0.06319	−0.3840	*p* < 0.001 *
Alfacalcidol (vs. monotherapy)	5.537 × 10^−3^	0.03271	0.0156	0.86571
Eldecalcitol (vs. monotherapy)	0.05585	0.02739	0.1889	0.04254 *
Model fit: R^2^ = 0.162, Adjusted R^2^ = 0.145, *n* = 249				

Values are from multivariable linear regression analyses. β indicates the unstandardized regression coefficient; SE(β), the standard error of β; stdβ, the standardized regression coefficient. Vitamin D co-therapy was treated as a three-group categorical variable and modeled using two dummy variables: eldecalcitol co-therapy (1 = yes, 0 = otherwise) and alfacalcidol co-therapy (1 = yes, 0 = otherwise), with SERM monotherapy (no active vitamin D) as the reference category. *p* values are two-sided. Abbreviations: BMD, bone mineral density; eGFR, estimated glomerular filtration rate; SERM, selective estrogen receptor modulator. *n* = 249; residual df = 243. Statistical significance was set at *p* < 0.05. * *p* < 0.05, and ** *p* < 0.001.

## Data Availability

The datasets used and/or analyzed during the current study are available from the corresponding author on reasonable request.

## Supplementary Materials

The following supporting information can be downloaded at: https://www.mdpi.com/article/10.3390/medicina62071220/s1. Table S1: Sensitivity analyses of BMD change ratios after additional adjustment for SERM type (bazedoxifene vs. raloxifene); Table S2: Baseline and 1-year BMD values and changes stratified by age group. BMD, bone mineral density; SERM, selective estrogen receptor modulator.

## Abbreviations

ACP	American College of Physicians
ANOVA	analysis of variance
BAP	bone-specific alkaline phosphatase
BMD	bone mineral density
DXA	dual-energy X-ray absorptiometry
eGFR	estimated glomerular filtration rate
FRAX	Fracture Risk Assessment Tool
NTX	type I collagen cross-linked N-telopeptide
PTH	parathyroid hormone
SERM	selective estrogen receptor modulator
STROBE	Strengthening the Reporting of Observational Studies in Epidemiology
