# Supplementary material for: Site-Specific Responses to SERM Treatment in Postmenopausal Osteoporosis: No Clear Age Attenuation in a Real-World Study
Source: Medicina (Kaunas). 2026 Jun 23;62(7):1220. doi: 10.3390/medicina62071220 (PMC13413720; doi:10.3390/medicina62071220)
Supplement: Supplementary file 1 [file medicina-62-01220-s001.zip › medicina-4310889-supplementary.pdf]

medicina

MDPI

Supplementary Table S1.

Sensitivity analyses including SERM type

Table S1. Multivariable linear regression analyses of factors associated with 1-year BMD change ratios (1-year/baseline) after SERM therapy, additionally adjusted for SERM type (bazedoxifene vs raloxifene).

n = 249. Lumbar spine model: $R^2 = 0.0595$ (adjusted $R^2 = 0.0362$). Femoral neck model: $R^2 = 0.1635$ (adjusted $R^2 = 0.1427$).

Table S1-1. Lumbar spine BMD change ratio (1-year/baseline)

Variable	βB	SE(β)	stdβ	t	df	p
Intercept	1.089	0.0417	—	—	—	—
Baseline age (years)	−0.00003	0.00043	−0.004	−0.066	242	0.947
Baseline eGFR	−0.00001	0.00018	−0.003	−0.039	242	0.969
Baseline lumbar spine BMD (L2–4)	−0.0880	0.02508	−0.226	−3.509	242	<0.001
Eldecalcitol (vs none)	0.01567	0.01352	0.114	1.159	242	0.247
Alfacalcidol (vs none)	−0.0040	0.01636	−0.002	−0.024	242	0.981
Bazedoxifene (vs raloxifene)	0.001246	0.00812	0.010	0.154	242	0.878

Table S1-2. Femoral neck BMD change ratio (1-year/baseline)

Variable	**β**	**SE(β)**	**stdβ**	**t**	**df**	**p**
Intercept	1.258	0.08546	—	—	—	—
Baseline age (years)	0.0002	0.00087	0.0159	0.254	242	0.799
Baseline eGFR	−0.0007	0.00037	−0.1209	−1.931	242	0.055
Eldecalcitol (vs none)	0.05623	0.02744	0.1902	2.050	242	0.041
Baseline femoral neck BMD	−0.4099	0.0642	−0.390	−6.383	242	<0.001
Alfacalcidol (vs none)	0.00470	0.0328	0.013	0.143	242	0.886
Bazedoxifene (vs raloxifene)	−0.0098	0.0167	−0.035	−0.589	242	0.556

β indicates the unstandardized regression coefficient; SE(β), the standard error; stdβ, the standardized regression coefficient. Active vitamin D co-therapy was modeled using two dummy variables (alfacalcidol and eldecalcitol), with no active vitamin D as the reference category. SERM type was coded as bazedoxifene = 1 and raloxifene = 0. Statistical significance was set at $p < 0.05$.

Supplementary Table S2.

(A) Lumbar spine (L2–4) BMD

Age group	n	Baseline mean	1-year mean	Δ (1-year − baseline), mean ± SD	p (paired t-test)
50–64 years	85	0.753	0.776	+0.023 ± 0.030	<0.001
65–74 years	112	0.784	0.805	+0.022± 0.049	<0.001
≥75 years	72	0.791	0.809	+0.017 ± 0.041	<0.001
**Total**	**269**	**0.776**	**0.797**	**+0.021 ± 0.042**	**<0.001**

(B) Femoral neck (Fneck) BMD

Age group	n	Baseline mean	1-year mean	Δ (1-year − baseline), mean ± SD	p (paired t-test)
50–64 years	85	0.570	0.582	+0.012 ± 0.046	0.017
65–74 years	112	0.562	0.570	+0.008 ± 0.053	0.094
≥75 years	72	0.550	0.560	+0.010± 0.064	0.178
Total	269	0.561	0.571	+0.010 ± 0.054	0.002
